# An Updated Meta-Analysis of Fatal Adverse Events Caused by Bevacizumab Therapy in Cancer Patients

**DOI:** 10.1371/journal.pone.0089960

**Published:** 2014-03-05

**Authors:** Hongxin Huang, Yayuan Zheng, Jianhong Zhu, Jingjing Zhang, Huapu Chen, Xinggui Chen

**Affiliations:** 1 Clinical Research Center, Affiliated Hospital of Guangdong Medical College, Zhanjiang, China; 2 Department of Pharmacology, Guangdong Medical College, Zhanjiang, China; Sudbury Regional Hospital, Canada

## Abstract

**Background:**

The risk of fatal adverse events (FAEs) due to bevacizumab-based chemotherapy has not been well described; we carried out an updated meta-analysis regarding this issue.

**Methods:**

An electronic search of Medline, Embase and The Cochrane Central Register of Controlled Trials was conducted to investigate the effects of randomized controlled trials on bevacizumab treatment on cancer patients. Random or fixed-effect meta-analytical models were used to evaluate the risk ratio (RR) of FAEs due to the use of bevacizumab.

**Results:**

Thirty-four trials were included. Allocation to bevacizumab therapy significantly increased the risk of FAEs; the RR was 1.29 (95% CI:1.05–1.57). This association varied significantly with tumor types (*P* = 0.002) and chemotherapeutic agents (*P* = 0.005) but not with bevacizumab dose (*P* = 0.90). Increased risk was seen in patients with non–small cell lung cancer, pancreatic cancer, prostate cancer, and ovarian cancer. However, FAEs were lower in breast cancer patients treated with bevacizumab. In addition, bevacizumab was associated with an increased risk of FAEs in patients who received concomitant agents of taxanes and/or platinum.

**Conclusion:**

Compared with chemotherapy alone, the addition of bevacizumab was associated with an increased risk of FAEs among patients with special tumor types, particularly when combined with chemotherapeutic agents such as platinum.

## Introduction

Bevacizumab, a humanized monoclonal antibody against the vascular endothelial growth factor (VEGF), has shown to be beneficial in the treatment of many types of metastatic cancers including metastatic colon cancer, renal cancer, non–small cell lung cancer (NSCLC), and breast cancer [1]–[5]. However, life-threatening side effects associated with the use of bevacizumab have been reported, including gastrointestinal (GI) perforation, non-healing wounds, hemorrhage, thromboembolic events, severe high blood pressure, infusion reactions, stroke, and heart problems [6], [7].

Fatal adverse events (FAEs) are defined as deaths that are linked to the use of a pharmaceutical agent [8]. A previous study using pooled analysis from 16 randomized controlled trials (RCTs), which included 10,217 patients total, indicated that bevacizumab, in addition to chemotherapy, was associated with an increased risk of FAEs when compared with chemotherapy alone [9]. This association varied significantly with chemotherapeutic agents but not with tumor types or bevacizumab dose. However, several meta-analyses, where FAEs were the secondary endpoint, showed conflicted results [10]–[14]. There are a couple of issues regarding the use of bevacizumab that have not been fully studied. Firstly, studies on the effect of bevacizumab on FAEs have been inconclusive so far. Secondly, because bevacizumab was associated with survival benefits in some trials, it means that patients in these trials treated with bevacizumab had more time to develop FAEs compared with controls; this potential bias may influence the overall results.

Considering the conflicting results of meta-analyses and the number of RCTs that have been published since then, we performed an updated systematic review and meta-analysis to evaluate the effect of bevacizumab on the occurrence of FAEs in cancer patients.

## Materials and Methods

### Search strategy

In accordance with PRISMA statement [15], we performed a literature search for the purpose of identifying RCTs. We searched the electronic databases Medline, Embase and The Cochrane Central Register of Controlled Trials up to August 2013. The search terms included “bevacizumab”, “Avastin”, and “cancer”. Conference abstracts from the American Society of Clinical Oncology held up to August 2013 containing terms such as bevacizumab and Avastin were also searched in order to identify relevant clinical trials, and original authors were contacted for possible unpublished data. We also searched for any additional studies in the reference lists of recent meta-analysis of bevacizumab treatment on cancer. For duplicate publications, only the most detailed articles were included. Our searches were limited to human trials and no language was restricted.

### Eligibility criteria

The search results were then screened on the basis of the following criteria.

1. *Types of studies*: Participants were chosen from either randomized Phase II or Phase III trials of patients with cancer.
2. *Interventions*: Participants were randomly assigned to treatment with bevacizumab or non-bevacizumab containing therapy.
3. *Outcome*: The number of FAEs was reported separately for the bevacizumab treatment group and the control group.

### Data extraction and quality assessment

Two statisticians independently extracted information from included studies using a standardized form; a third statistician verified them. Information collected included: first author, publishing year, trial phase, sample size, treatment arms, median treatment duration, dosage of bevacizumab, and the number of FAEs. Quality assessment of included studies was conducted by two independent researchers through collecting data on sources of systematic bias using the Jadad score [16]. Methodological features most relevant to the control of bias were examined, including: random sequence generation, allocation concealment, blinding of participants and personnel, blinding of outcome assessment, and incomplete outcome data [16].

### Data analysis

Data analyses were performed using Review Manager (Version 5.1). For the calculation of incidence, the number of patients with FAEs and the sample size of each group were extracted from the selected trials; and the proportion of patients with FAEs and 95% confidence interval (CI) were derived for each study. The Mantel-Haenszel method was used to calculate RR and 95% CI of FAEs in patients assigned to bevacizumab group versus control group in the same study. We assessed the statistical heterogeneity among studies included in the meta-analysis with Cochrane's Q statistic, and quantified inconsistency with the I^2^ statistic [100%×(Q - df)/Q]. When I^2^ statistic was greater than 50%, suggesting substantial heterogeneity, a random effects model was used, whereas a fixed effects model was used when I^2^ statistic was less than 50% [17], suggesting that heterogeneity could be neglected. The presence of publication bias was evaluated by using the Begg and Egger tests. A p-value less than 0.05 was considered to be statistically significant.

We performed four subgroup analyses: (1) to estimate effects separately according to the type of tumor; (2) to estimate effects separately for low-dose (2.5 mg/kg per wk) and high-dose (5 mg/kg per wk); (3) to estimate effects separately according to chemotherapeutic agent; (4) to estimate effects separately according to median progression-free survival (PFS). The summary RRs for subgroups were compared using a standard chi-squared test.

## Results

### Search results

**Figure 1:**
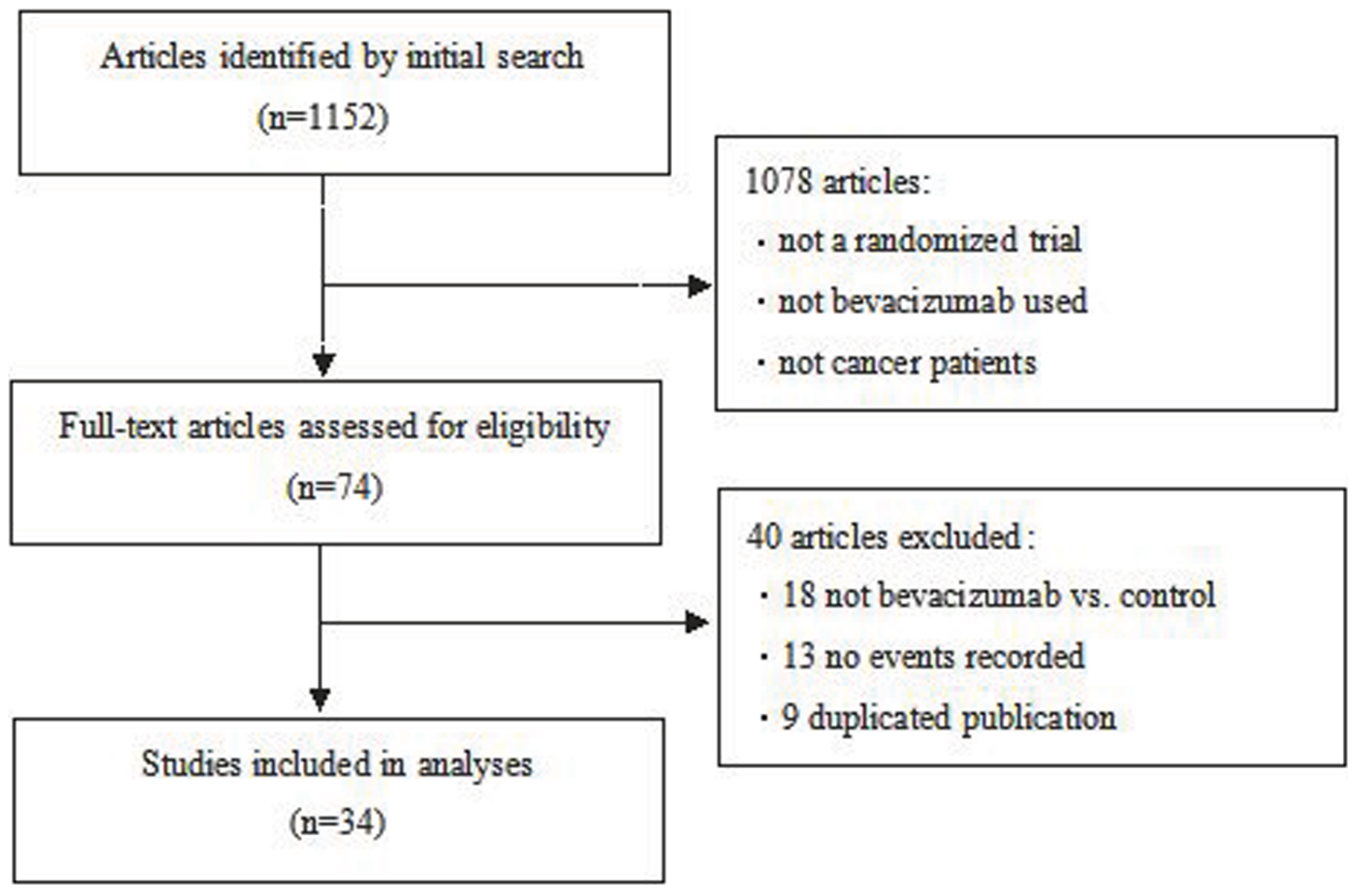
Study flow diagram.

A total of 1,152 unique titles and abstracts were found from initial searches of the electronic database. We applied the inclusion/exclusion criteria to filter out 1,078 titles and abstracts. An additional 40 articles were further excluded after a full-text review. Our final database therefore included 34 trials (8 phase 2 and 26 phase 3) comprising 25,424 participants [1]–. The details of study selection flow are described in Figure 1.

### Study characteristics

The pooled population for these analyses included 25,424 patients, of whom 13,656 were randomly assigned to receive bevacizumab and 11,768 were randomly assigned to control groups (Table 1). Patients included in those trials followed the eligibility criteria defined by each unique trial and generally included patients with good performance status. Ten trials were carried out in patients with colorectal cancer, ten in patients with breast cancer, seven in patients with non-small-cell lung cancer, three in patients with ovarian cancer, each two in renal cell cancer and pancreatic cancer patients, one each in prostate cancer, gastric cancer, extensive-stage small-cell lung cancer (SCLC) respectively. The co-therapy agents administered varied with tumor types. In addition, 5,377 (39.4%) of the bevacizumab-treated patients received it at a dose intensity of 2.5 mg/kg per week, and 8,279 (60.6%) received it at 5 mg/kg per week. Examination of individual trial design revealed that randomized treatment allocation sequences were generated in all included trials; 14 trials were double-blinded. The median Jadad score was 3 (range = 2–4) and quality assessment suggested that the overall study quality was fair.

**Table 1:** Characteristics of studies included in primary analysis.

Study	Trial Phase	Tumor Type	Concurrent Treatment	No in intervention/control*	Bevacizumab dose, mg/kg per week	Jadad score
Bennouna, 2013	III	Colorectal cancer	bolus fluorouracil or capecitabine plus oxaliplatin or irinotecan	401/409	2.5	3
Giantonio, 2007	III	Colorectal cancer	Oxaliplatin, fluorouracil, leucovorin	287/285	5	2
de Gramont, 2012	III	Colon cancer	Fluorouracil, leucovorin, oxaliplatin	1145/1126	2.5	3
Guan, 2011	III	Colorectal cancer	Irinotecan, leucovorin bolus, 5-fluorouracil	141/70	2.5	3
Hurwitz, 2004	III	Colorectal cancer	Irinotecan, leucovorin, bolus fluorouracil	393/397	2.5	3
Kabbinavar, 2003	II	Colorectal cancer	Fluorouracil, leucovorin	67/35	2.5 or 5	2
Kabbinavar, 2005	II	Colorectal cancer	Bolus fluorouracil, leucovorin	100/104	2.5	3
Saltz, 2008	III	Colorectal cancer	Oxaliplatin, fluorouracil, and folinic or capecitabine and oxaliplatin	694/675	2.5	4
Tebbutt, 2010	III	Colorectal cancer	Capecitabine	157/156	2.5	3
Bear, 2012	III	Breast cancer	capecitabine or gemcitabine plus docetaxel	595/596	5	2
Brufsky, 2011	III	Breast cancer	Docetaxel or gemcitabine or capecitabine or vinorelbine	458/221	5	4
Cameron, 2013	III	Breast cancer	Anthracycline or taxane	1288/1271	5	3
Gianni, 2013	III	Breast cancer	Docetaxel, trastuzumab	215/206	5	2
Martin, 2011	II	Breast cancer	Paclitaxel	96/89	5	3
Miles, 2010	II	Breast cancer	Docetaxel	499/231	2.5 or 5	3
Miller, 2005	III	Breast cancer	Capecitabine	229/215	5	2
Miller, 2007	III	Breast cancer	Paclitaxel	365/346	5	3
Robert, 2011	III	Breast cancer	Docetaxel, capecitabine, anthracycline	817/403	5	3
Herbst, 2007	II	NSCLC	Docetaxel, pemetrexed	39/42	5	3
Herbst, 2011	III	NSCLC	Erlotinib	313/313	5	4
Johnson, 2004	II	NSCLC	Paclitaxel, carboplatin	66/32	2.5 or 5	3
Niho, 2012	II	NSCLC	Carboplatin, paclitaxel	119/58	5	2
Reck, 2009	III	NSCLC	Cisplatin, gemcitabine	659/327	2.5 or 5	4
Sandler, 2006	III	NSCLC	Carboplatin, paclitaxel	427/440	5	3
Aghajanian, 2012	III	Ovarian, peritoneal, fallopian tube cancer	Gemcitabine, carboplatin	242/242	5	4
Burger, 2011	III	Ovarian cancer	Carboplatin, paclitaxel	608/601	5	3
Perren, 2011	III	Ovarian cancer	Carboplatin, paclitaxel	745/753	2.5	3
Escudier, 2007	III	Renal cell carcinoma	interferon alfa	337/304	5	4
Rini, 2010	III	Renal cell carcinoma	Interferon alfa	362/347	5	2
Van Cutsem, 2009	III	Pancreatic cancer	Gemcitabine, erlotinib	296/287	2.5	4
Kindler, 2010	III	Pancreatic cancer	Gemcitabine	277/263	2.5	4
Kelly, 2012	III	Prostate cancer	Docetaxel, prednisone	504/505	5	3
Ohtsu, 2011	III	Gastric cancer	fluoropyrimidine, cisplatin	386/381	2.5	4
Spigel, 2011	II	SCLC	Cisplatin or carboplatin plus etoposide	51/47	5	4

*Number of patients for safety analysis; NSCLC, non– small cell lung cancer; SCLC, small cell lung cancer.

### Incidence of FAEs

There were 241 FAEs reported for 13,656 patients who received bevacizumab (Table 2). The highest incidence (6.06%; 95% CI: 0.99%–11.12%) was observed in a pancreatic cancer trial. The lowest incidence (0.69%; 95% CI: 0.28%–1.09%) was seen in the trials of patients with breast cancer. Using a random-effects model we found that the summary incidence of FAEs in patients receiving bevacizumab was 1.48% (95% CI: 1.12%–1.83%).

**Table 2:** Risk ratio of fatal adverse events by subgroup.

Subgroup	Studies n	Bevacizumab arm			Control arm			Risk Ratio (95% CI)	I^2^ value (%)	P value	
		No. of events	No. of patients	Incidence (%)	No. of events	No. of patients	Incidence (%)			RR	Group difference
Overall	34	241	13656	1.48	149	11768	0.93	1.29 (1.05, 1.57)	16	0.01	NA
Dose											0.90
2.5 mg/kg per wk	15	116	5377	2.33	88	5246	1.52	1.29 (0.98, 1.69)	0	0.07	
5 mg/kg per wk	23	125	8279	1.24	83	7147	0.84	1.25 (0.96, 1.64)	20	0.10	
Tumor type											0.002
Colorectal cancer	9	49	3619	1.51	35	3257	0.89	1.29 (0.84, 1.99)	0	0.24	
Breast cancer	9	38	4562	0.69	37	3578	0.85	0.61 (0.39, 0.95)	0	0.03	
NSCLC	6	59	1662	3.06	18	1212	1.03	1.88 (1.15, 3.07)	34	0.01	
Ovarian cancer	3	19	1600	0.95	8	1587	0.42	2.35 (1.03, 5.33)	0	0.04	
Renal cell cancer	2	11	699	1.45	11	651	1.55	0.92 (0.40, 2.11)	0	0.84	
Pancreatic cancer	2	36	573	6.06	19	550	3.20	1.83 (1.07, 3.14)	0	0.03	
Prostate cancer	1	20	504	3.97	6	505	1.19	3.34 (1.35, 8.25)	NA	0.009	
Gastric cancer	1	7	386	1.81	12	381	3.15	0.58 (0.23, 1.45)	NA	0.24	
SCLC	1	2	51	3.92	3	47	6.38	0.61 (0.11, 3.52)	NA	0.58	
Chemotherapeutic agents									0.005		
With platinum	13	105	6069	1.53	53	5367	0.68	1.54 (1.11, 2.13)	16	0.009	
Without platinum	21	136	7587	1.54	96	6401	1.24	1.15 (0.89, 1.48)	16	0.29	
With taxanes	15	95	6266	1.35	45	5493	0.56	1.60 (1.14, 2.25)	34	0.007	
Without taxanes	20	146	7390	1.72	104	6275	1.34	1.14 (0.89, 1.46)	0	0.29	
Platinum and taxanes	5	42	1965	2.08	10	1884	0.41	3.57 (1.83, 7.00)	0	0.0002	
Median PFS									0.45		
Similar	5	32	3565	0.91	20	3492	0.47	1.54 (0.89, 2.69)	31	0.12	
Significant different	30	209	10091	1.66	136	8507	1.14	1.23 (1.00, 1.52)	13	0.05	

NSCLC, non– small cell lung cancer; SCLC, small cell lung cancer; RR, risk ratio; PFS, progression-free survival; NA, not applicable.

### Risk ratio of FAEs

**Figure 2:**
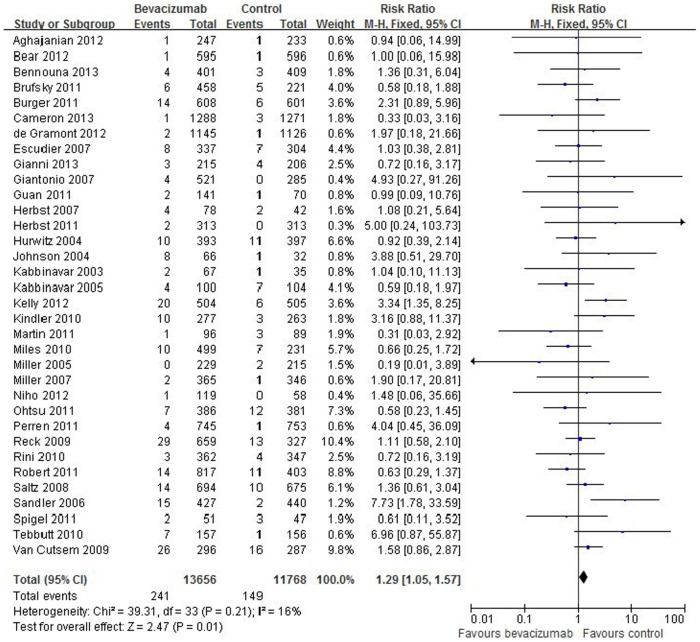
Risk ratio of fatal adverse events in cancer participants treatment with bevacizumab compare with control.

In order to assess the contribution of bevacizumab in the development of FAEs, we calculated the overall RR of FAEs. The overall RR of FAEs for patients treated with bevacizumab compared to that of the control group was 1.29 (95% CI:1.05–1.57), a statistically significant finding (P = 0.01) with insignificant heterogeneity (I^2^ = 16%) (Figure 2).

### Subgroup analysis according to tumor type

We carried out a subgroup analysis to determine whether the tumor type had an influence on the RR of FAEs with bevacizumab. Significantly increased risk of FAEs was seen in the following tumor types: NSCLC (RR, 1.88; 95% CI, 1.15–3.07), pancreatic cancer (RR, 1.83; 95% CI, 1.07–3.14), prostate cancer (RR, 3.34; 95% CI, 1.35–8.25), ovarian cancer (RR, 2.35; 95% CI, 1.03–5.33). Significantly decreased risk of FAEs was seen in breast cancer (RR, 0.61; 95% CI, 0.39–0.95). RR of FAEs varied significantly by tumor types (*P* = 0.002) (Table 2).

### Subgroup analysis according to dose regimen

To investigate whether dose regimens have the potential to alter the association of bevacizumab with risk of FAEs, we performed a subgroup analysis stratified according to dose class such as high-dose group (5 mg/kg per wk) and low-dose group (2.5 mg/kg per wk). Neither the low dose nor the high dose administration was associated with an increased risk of FAEs. For high-dose group, the RR of FAEs for patients treated with bevacizumab compared with that for control was 1.29 (95% CI: 0.98–1.69). For low-dose group, the RR was 1.25 (95% CI: 0.96–1.64). No statistically significant difference was observed among dose regimens (*P* = 0.90) (Table 2).

### Subgroup analysis according to chemotherapy regimen

To determine whether the type of chemotherapeutic agent may alter the association of bevacizumab with risk of FAEs, we performed a subgroup analysis stratified according to chemotherapeutic agents. We divided all the trials into two arms: co-therapy with platinum (cisplatin, carboplatin, or oxaliplatin) and co-therapy without platinum. The RR of bevacizumab with platinum was 1.54 (95% CI: 1.11–2.13) vs 1.15 (95% CI: 0.89–1.48) for non-platinum. We further divided all the trials into two additional arms: taxanes (paclitaxel or docetaxel) and co-therapy without taxanes. The RR of bevacizumab with taxanes was 1.60 (95% CI: 1.14–2.25) vs 1.14 (95% CI: 0.89–1.46) for non-taxanes. Significant increased risk was seen in bevacizumab co-therapy with platinum and taxanes, the RR was 3.57 (95% CI: 1.83–7.00). Statistically significant differences were observed among chemotherapeutic classes (*P* = 0.005) (Table 2).

### Subgroup analysis according to median progression-free survival

We investigated whether the duration of use of bevacizumab led to an increased risk of high-grade VTE. We used median PFS as a surrogate for duration of treatment, and performed a subgroup analysis stratified according to PFS. Median PFS was similar between bevacizumab and control vs. median when PFS was significant different between bevacizumab and control. The RR for the similar median PFS between bevacizumab and control was 1.54 (0.89, 2.69) vs 1.23 (1.00, 1.52) for the significantly different median PFS between bevacizumab and control. The RR of FAEs did not vary significantly by the difference of median PFS between bevacizumab group and control group (P = 0.45) (Table 2).

### Risk of specific fatal adverse events

Individual specified and non-specified causes of FAEs are listed in table 3. As shown, 89 FAEs within the bevacizumab group and 34 FAEs within the control group were reported specified. Of the reported causes of FAEs, the rates of hemorrhage, pulmonary embolism, neutropenia, gastrointestinal tract perforation, and cerebrovascular accident were numerically higher on the bevacizumab treatment arms. Other causes of deaths were infrequent and occurred in isolation.

**Table 3:** Fatal adverse events by specific type.

Fatal adverse event	Events on bevacizumab arms	Events on control arms
Hemorrhage	32	1
Pulmonary hemorrhage	17	1
Gastrointestinal hemorrhage	11	0
Pulmonary embolism	9	4
Neutropenia	7	2
Gastrointestinal tract perforation	8	2
Cerebrovascular accident	7	2
sepsis	3	6
Cardiac ischemia/infarction	1	3
sudden death	2	0
Other	20	14
Not specified	152	115
Total	241	149

### Publication bias

No evidence of publication bias was detected for the RR of FAEs in this study by either Begg or Egger's test (RR of FAEs: Begg's test P = 0.423; Egger's test P = 0.660).

## Discussion

We performed an updated and systematic review and meta-analysis of evidence regarding the risk of FAEs in cancer patients who were treated with bevacizumab. Our results demonstrated that compared with chemotherapy alone, the addition of bevacizumab was associated with an increased risk of FAEs.

Our results showed that the most significant risk of FAEs was in patients with prostate cancer and NSCLC, as reported by others [14]. Our analysis also suggested that no significant difference was seen in colorectal cancer patients [12], in concordance with another recently published meta-analysis. A previous meta-analysis indicated that no significant relationship was found between bevacizumab and FAEs [11], but FAEs were even lower for breast cancer patients treated with bevacizumab in the present study. In contrast to Ranpura et al [9], we found that the RR of FAEs associated with bevacizumab varied significantly with tumor types (P = 0.002). It may indicate that a tumor-specific interaction between bevacizumab and tumor type in terms of toxicity cannot be excluded, and that bevacizumab-related toxicity may thus have contributed to the negative outcome of studies in NSCLC, pancreatic cancer, prostate cancer, and ovarian cancer. For instance, in a phase II trial treating patients who presented with squamous cell histology with a combination therapy of chemotherapy and bevacizumab four out of thirteen patients ended up with life-threatening or fatal hemoptysis [34].

Ranpura et al [9] found that the association of bevacizumab with FAEs was statistically significant following higher dosing of bevacizumab (5.0 mg/kg per week) for patients with advanced cancer. That finding was not confirmed in our analysis. Our results indicated that the association of bevacizumab with FAEs was not statistically significant in the subgroup of both higher dose of bevacizumab (5.0 mg/kg per week) or lower dose of bevacizumab (2.5 mg/kg per week). Furthermore, there was no significance between the high and low doses of bevacizumab (*P* = 0.90). This indicates that dose regimens may not alter the association of bevacizumab with risk of FAEs.

Our results confirmed the previous study by Ranpura et al [9], which also found significant difference in risk of FAEs with bevacizumab among different chemotherapeutic (*P* = 0.005). This may be because treatment with bevacizumab, in combination with platinum or taxanes, resulted in more toxic effects than bevacizumab combined with other agents. A RCT comparing bevacizumab plus paclitaxel with bevacizumab plus capecitabine suggested that the proportion of patients discontinuing chemotherapy because of adverse events was twice as high with paclitaxel compared with capecitabine [47]. Another study compared the efficacy and safety of bevacizumab when combined with several standard chemotherapy regimens and found that grade 3 to 5 adverse events were higher in the bevacizumab plus taxane arms as compared to bevacizumab plus capecitabine or anthracycline arms [33].

Patients in some trials stayed on treatment with bevacizumab for much longer than control groups because bevacizumab improved PFS. Thus, it is possible that patients in these trials treated with bevacizumab have more time to develop FAEs than controls [48]. Therefore, we analyzed three trials [19], [30], [43] in which bevacizumab was not associated with prolonged time to progression and two other trials where FAEs were reported during the chemotherapy phase rather than the extended therapy phase [27], [39]. We found that the RR of FAEs with bevacizumab from these five trials was 1.54 (0.89, 2.69) vs 1.23 (1.00, 1.52) compared to the other thirty trials, where bevacizumab was associated with significantly prolonged time to progression. Thus, it appears that potential biases due to a prolonged time to progression associated with bevacizumab may not have an effect on the risk of FAEs.

Similar to other meta-analyses, our review has several limitations. Firstly, in the case of patient selection criteria, classes of chemotherapeutic agents vary greatly between studies, which is likely to produce certain effects on the final outcome. Secondly, some studies did not clearly differentiate between disease-related and non-disease-related fatal events. It is possible that some of the FAEs were not treatment related, which is likely to produce inaccuracies in outcome reporting. Thirdly, all of the included studies were conducted in patients with adequate organ function at study entry whereas the association between bevacizumab and FAEs in general patient population and patients with organ dysfunction are still inconclusive. Lastly, as FAEs were not specified in most trials, we could not fully characterize the cause of FAEs.

In conclusion, the use of bevacizumab therapy was associated with a small but significant increase in the risk of fatal drug-related events, especially when combined with chemotherapeutic agents such as platinum (cisplatin, carboplatin, or oxaliplatin) and taxanes (paclitaxel or docetaxel). The risk ratio of FAEs associated with bevacizumab varied significantly with tumor types but not with bevacizumab dose. Patients with NSCLC, pancreatic cancer, prostate cancer, and ovarian cancer had significant increased risk of FAEs. Moreover, FAEs was lower for breast cancer patients treated with bevacizumab. Based on our study, in combination with previous meta-analyses, we strongly suggest that all patients treated with bevacizumab should be monitored carefully for bleeding, gastrointestinal tract perforation, pulmonary embolism, and cerebrovascular accident.

## Supporting Information

Checklist S1 **PRISMA Checklist.**
(DOC)
